# Whole-exome sequencing of 79 xenografts as a potential approach for the identification of genetic variants associated with sensitivity to cytotoxic anticancer drugs

**DOI:** 10.1371/journal.pone.0239614

**Published:** 2020-09-28

**Authors:** Chihiro Udagawa, Yasushi Sasaki, Yasuhiro Tanizawa, Hiroshi Suemizu, Yasuyuki Ohnishi, Yasukazu Nakamura, Takashi Tokino, Hitoshi Zembutsu

**Affiliations:** 1 Department of Genetic Medicine and Services, National Cancer Center Hospital, Tokyo, Japan; 2 Biology, Department of Liberal Arts and Sciences Center for Medical Education, Sapporo Medical University, Sapporo, Japan; 3 Department of Informatics, National Institute of Genetics, Mishima, Japan; 4 Laboratory Animal Research Department, Central Institute for Experimental Animals, Kawasaki, Japan; 5 Department of Medical Genome Sciences, Research Institute for Frontier Medicine, Sapporo Medical University, Sapporo, Japan; 6 Department of Clinical Genomics, National Cancer Center Research Institute, Tokyo, Japan; Ohio State University Wexner Medical Center, UNITED STATES

## Abstract

Chemotherapy response remains unpredictable in most patients with cancer. In this study, we performed whole-exome sequencing of 79 cancer xenografts derived from human cancer tissues to identify genetic predictors of chemosensitivity to nine cytotoxic anticancer drugs. Xenografts were harvested from 12 organs with cancer and implanted into nude mice. The mice were exposed to one of nine cytotoxic anticancer drugs (5-fluorouracil, nimustine, adriamycin, cyclophosphamide, cisplatin, mitomycin C, methotrexate, vincristine, and vinblastine) to assess the correlation between chemosensitivity response and variant allele frequency. We found 162 candidate variants that were possibly associated with chemosensitivity to one or more of the nine anticancer drugs (*P* < 0.01). In a subgroup analysis of breast and gastric cancer xenografts, 78 and 67 variants, respectively, were possibly associated with chemosensitivity. This approach may help to contribute to the development of personalized treatments that may allow for the prescription of optimal chemotherapy regimens among patients with cancer.

## Introduction

Cancer is a global health concern, with approximately 18.1 million new cases and 9.6 million deaths in 2018 [1]. Currently, most cancers are treated by surgery, radiation therapy, and/or chemotherapy [2,3]. Chemotherapy remains a gold standard for the treatment of blood cancers, such as leukemia and lymphoma [4–6], unresectable or metastatic cancers [7,8], and solid tumors, such as lung, breast and colorectal cancers [9–11]. Despite an improved understanding of cancer biology and the development of molecular targeted therapy and immunotherapy for select patients [12,13], chemotherapy still plays a primary role in cancer treatment regimes. Indeed, chemotherapy regimens have improved considerably, now taking into consideration the organ of origin, histological appearance, and stage of progression. Yet, these improvements aside, chemotherapeutic efficacy still varies between individuals [14] and is often complicated by toxic reactions, including nausea, tiredness, diarrhea, and hair loss [14,15], causing physical and mental distress and decreased patient quality of life. As such, it is becoming increasingly important to identify effective treatments with fewer toxic side effects as a first-line therapy for each patient. Several recent studies have sought to establish diagnostic methods for predicting chemosensitivity to cytotoxic anticancer drugs before treatment is undertaken. However, clinically useful genetic markers have yet to be developed [16–20].

Patient-derived xenograft (PDX) models have been established for many types of tumors and have emerged as powerful tools for predicting drug efficacy and for understanding tumor characteristics. With PDX models, fresh human tissue is directly implanted into immuno-compromised mice. These models retain the heterogeneity of the original patient tumors and thus allow for tests, predominantly to examine the efficiency of anticancer drugs [21].

Next-generation sequencing technologies have also been developed in recent years, exposing tumor genomic profiles and facilitating the detection of low frequency variants and other genetic mutations that could not otherwise be uncovered by conventional methods [22,23]. Indeed, several studies have reported associations between such genetic mutations in tumors and clinical outcomes [24–26]. Guided by these reports, we hypothesized that genetic variants within tumors, including low frequency, rare variants, may underpin patient responses to cytotoxic anticancer drugs, such as chemosensitivity and chemoresistance. To this end, we performed whole-exome sequencing of DNA samples taken from 79 human cancer xenografts prepared from 12 different organs. These xenografts were implanted in mice and treated with one of nine cytotoxic anticancer drugs. We assessed correlations between the chemosensitivities of the xenografts to nine anticancer drugs and the variant allele frequencies (VAFs) as a potential approach to identify variants that may be predictive of drug response.

## Materials and methods

### Animals and tumor xenograft model

Previously, a total of 79 human tumor tissues were obtained aseptically during surgery or at autopsy across 13 hospitals in Japan [27]. The samples included 12 breast cancers, 12 gastric cancers, 10 neuroblastomas, 10 non-small-cell lung cancers, 7 gliomas, 6 pancreatic cancers, 5 colon cancers, 5 choriocarcinomas, 4 small-cell lung cancers, 4 hematopoietic cancers, 3 ovarian cancers, and 1 osteosarcoma. These xenografts were separately transplanted into athymic BALB/c-nu/nu mice (Clea Japan, Inc, Tokyo, Japan) and maintained by serial subcutaneous transplantation of 2×2×2 mm fragments into the flank once a month, as described previously [27]. Microbiological monitoring of the tumor-bearing nude mice was performed for bacteria (e.g., *Pasteurella pneumotropica* and *Mycoplasma pulmonis*), viruses (e.g., mouse adenovirus and mouse hepatitis virus), and parasites (e.g., *Giardia muris* and *Spironucleus muris*) by culture, serological, or microscopic examinations [27]. Furthermore, histological examination and isozyme testing were carried out to assess for the risk of cross-contamination among the tumor lines, or cross-contamination between human tumor xenografts and a mouse tumor appearing at the inoculation site of the xenograft during passaging [27]. Tumor-bearing mice were euthanized with deep anesthesia followed by cervical dislocation. To minimize discomfort, euthanasia was performed quickly. Tumors were excised from the euthanized mice, and a piece of the tumor tissue was implanted into another mouse using a transplantation needle. All handling of mice was carried out in a gentle to minimize animal suffering and distress. The general conditions of the mice such as appetite and respiratory conditions were monitored every 2 or 3 days after transplantation, and the size of the tumor was measured twice a week. Mice were housed in a controlled temperature of 23±1°C and relative humidity 50–70%, with ad libitum access to food and water. All animal experiments were performed in accordance with the guidelines of the Central Institute for Experimental Animals.

### Anticancer drugs

The chemosensitivity tests on the xenograft model in this study were performed more than 20 years ago [27]. We chose nine cytotoxic anticancer drugs that could be classified into different categories based on their mechanism of action: 5-fluorouracil (5FU; Sigma-Aldrich; Merck KGaA, Darmstadt, Germany), nimustine (ACNU; Daiichi Sankyo Co., Ltd., Tokyo, Japan), adriamycin (ADR; Kyowa Hakko Bio Co., Ltd., Tokyo, Japan), cyclophosphamide (CPM; Shionogi & Co., Ltd., Osaka, Japan), cisplatin (DDP; Sigma-Aldrich), mitomycin (MMC; Kyowa Hakko Bio), methotrexate (MTX; Wyeth Lederle Japan, Ltd., Tokyo, Japan), vincristine (VCR; Shionogi & Co), and vinblastine (VLB; Shionogi & Co). These drugs have been used as a standard of care for cancer for over 20 years, and some of these drugs remain a standard of care. All of the drugs were dissolved in sterile 0.85% NaCl containing 1% mannitol (Wako Pure Chemical Industries, Ltd., Osaka, Japan).

### Chemosensitivity analysis

A total of 7,900 mice were purchased from Japan CLEA Inc. (Tokyo, Japan) and used in this study. Each anticancer drug was administered individually at the maximum tolerated dose (MTD) to nude mice bearing human cancer xenografts (*n* = 6 mice per group), because this dose could clearly distinguish responders from non-responders for each drug. The MTD for each drug was as described previously [27]: 6.7 mg/kg MMC, 260 mg/kg CPM, 48 mg/kg ACNU, 10 mg/kg DDP, 12 mg/kg ADR, 1.6 mg/kg VCR, 11 mg/kg VLB, 19 mg/kg 5-FU, 15 mg/kg MTX. 5-FU and MTX were administered once a day for 5 days whereas all other drugs were administered once. The control groups did not receive any treatment (6 mice per xenograft). Each of the 79 xenografts was treated with nine drugs over the course of the experiment, with 6 mice bearing xenografts used to test each drug. Two to four drugs were tested as part of a single cohort for each xenograft; along with 6 control mice, this equated to 18 to 30 mice at one time. In addition, 4 spare mice for each drug were prepared. This meant that up to 50 mice were used for each cohort. Mice were sacrificed by cervical dislocation at 21 days after administration of the drug or when the tumor volume reached 250 mm^3^ (humane endpoint criteria). The next cohort of mice were then acquired, and the same protocols were followed for housing and treatment. For a single xenograft, it took about 1.5 months to test each of the 9 drugs. Given that there were 79 xenografts in total, this part of our experimental procedures was carried out over approximately 10 years.

Chemosensitivity was calculated as the relative tumor volume in the treated mice (T) compared with the control (C) using the mean values measured on day 14, as described previously (T/C [%]) [27]. Tumor volume (mm^3^) was calculated using the following formula: 0.5 × major diameter × minor diameter^2^.

### Ethics statement

All animal studies were approved by the Institutional Committee of Central Institute for Experimental Animals, and carried out as per published protocols [27]. The establishment of PDX models and chemosensitivity testing of these xenografts were performed between 1981 and 1991. These studies were performed before enforcement of the Ethical Guidelines for Human Genome/Gene Analysis Research in Japan. Therefore, acquisition of agreement of patients for the use of their tumor was not obligated at the time. Furthermore, these xenografts are publicly available resources, and chemosensitivity data of them were published in 1996 [27]. Therefore approval from ethics committee is not necessary for this study.

### Sample preparation and whole-exome sequencing

Tumor genomic DNA was extracted from 79 xenografts using the QIAmp DNA Mini kit (QIAGEN, Hilden, Germany), according to the manufacturer’s protocol and as previously described [28]. Exome enrichment and library preparation were performed using Ion AmpiSeq Exome RDY Kit PI v3, which targets >97% of consensus coding sequences (CCDS) with 5-bp padding around exons, and Ion Xpress Barcode Adapters (Thermo Fisher Scientific, Inc.). Pooled barcoded libraries were subsequently conjugated with sequencing beads by emulsion PCR and enriched using the Ion PI Hi-Q Chef kit and Ion Chef (Thermo Fisher Scientific, Inc.). Sequencing of templates was performed with 2 samples per Ion PI Chip V3 using the Ion Proton system (Thermo Fisher Scientific, Inc.), according to the manufacturer’s protocols.

### Variant calling

To avoid false-positive results, we removed reads derived from the mouse genome, as follows. Sequencing reads were aligned to the human genome build 19 (hg19) and two mouse genomes C57BL/6J (mm10, NCBI accession number: GCA_000001635.26) and BALB/c (GCA_001632525.1) using the Torrent Mapping Alignment Program (ver 3.0.1, Thermo Fisher Scientific, Inc.). Reads aligned to the mouse genomes with higher alignment score than to the human genome were considered to be contamination from the host mouse and were removed from subsequent analyses. The Torrent Variant Caller plugin (ver 5.10.1, Thermo Fisher Scientific, Inc.) was used to identify variants. The parameter file, optimized for somatic mutations with low stringency criteria, was obtained from the software vendor. Variants were annotated by ANNOVAR (ver. 2018-04-16) [29] using the following reference databases: RefSeq Gene (refGene); LJB non-synonymous variants annotation (dbnsfp35a); dbSNP version 150 (avsn150); the 1000 Genome Project (1000g2015aug_eas); Clinvar version 20190305 (clinvar_20190305); COSMIC Release v88 (cosmic88); segmental duplication region (genomicSuperDups); transcription factor binding site (tfbsConsSites); Human Genetic Variation Database version 2.3 [30]; 3.5K Japanese individuals allele frequency panel (3.5KJPNv2) [31]. Variants were filtered and excluded if they: (i) had a quality score < 30; (ii) had segmental duplication or repeat regions identified by Repeat Masker or Tandem Repeats Finder; (iii) were found in homopolymer regions or multi-allelic sites; (iv) were previously detected in 3.5KJPNv2 or Human Genetic Variation Database (HGVD).

### Statistical analysis

The correlations between variant allele frequencies (VAFs) and drug sensitivities were assessed using Spearman correlation tests. Significance after Bonferroni correction for multiple testing was *P* = 1.11 x 10^−6^ (*P* < 0.05; 44,875 variants). Statistical tests were conducted using Microsoft Excel 2016 (Microsoft Corporation, Redmond, WA, USA).

## Results

### Identification of variants associated with chemosensitivity

Whole-exome sequencing was used to identify genetic variants associated with chemosensitivity to one or more of nine cytotoxic anticancer drugs (MMC, CPM, ACNU, DDP, ADR, VER, VLB, 5FU, and MTX). Drugs were administered to 79 PDX models of cancers prepared from 12 different human tissues. Between 30,012 and 45,638 variants were detected for each xenograft, with a coverage of 62 to 249 (mean; 37,921 variants, with depth of 138×). Variants were filtered using an in-house program (see Materials and methods), leaving a total of 44,875 variants for correlation analysis. Chemosensitivity was calculated as T/C and the variants whose allele frequency was higher in xenografts with lower T/C as were defined as ‘chemosensitive variants’ and variants whose allele frequency were higher in xenografts with higher T/C as ‘chemoresistant variants’.

**Fig 1:**
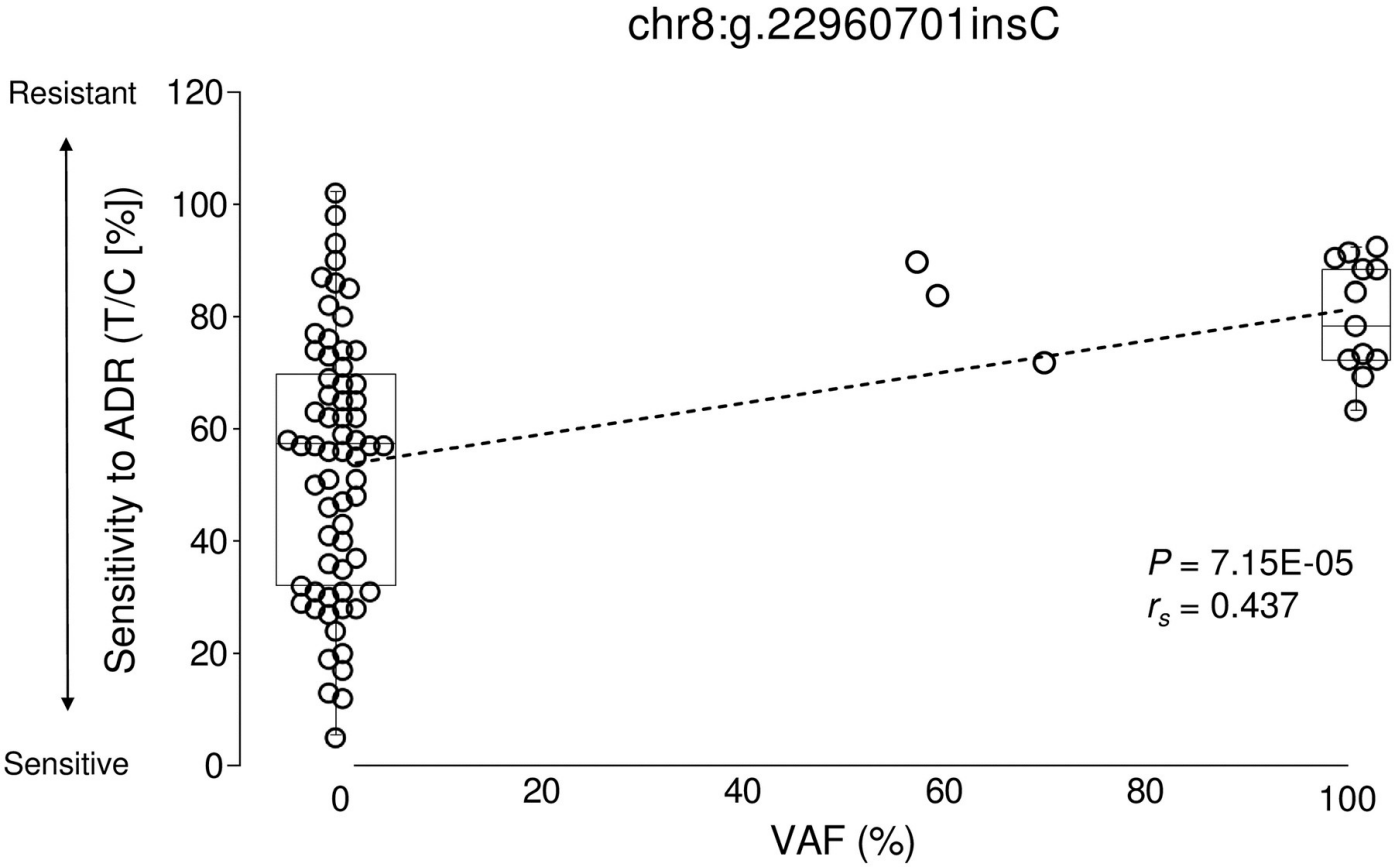
Correlation between variant chr8:g.22960701insC and chemosensitivity to ADR. Chemosensitivity to ADR is represented by relative tumor volume of treated mice (T) with respect to that of the control mice (C). Xenografts with a higher VAF exhibited a poorer response to ADR than those with a lower VAF.

Although no variants reached a significance level of *P* < 1.11 × 10^−6^ (see Materials and methods), we observed variants showing *P* < 0.01 (7.15 × 10^−5^ < *P* < 9.97 × 10^−3^; Tables 1–9). The variant (chr8:g.22960701 insC) with the highest significance (lowest *P* value) was associated with chemosensitivity to ADR, and was located on two overlapping genes: uncharacterized LOC254896 (*LOC254896*) and TNF receptor superfamily member 10c (*TNFRSF10C*) (*P* = 7.15 × 10^−5^, *r*_*s*_ = 0.437; Table 3, Fig 1). As presented in Fig 1, particular to this variant, xenografts with higher VAFs had poorer responses to ADR than those with lower VAFs. This may suggest that variant chr8:g.22960701 insC may be associated with resistance to ADR.

**Table 1:** Variants associated with chemosensitivity to 5FU (P < 0.01), as identified among 79 xenografts.

Drug	Chr	Position^a^	rsID^b^	Gene	Allele Ref./Variant	Location	Amino acid change	Prediction of functional effect		*P* value	*r*_*s*_^c^
								SIFT	PolyPhen2		
5FU	4	170428839	-	*NEK1*	-/A	intron				1.76E-03	-0.355
	19	57840351	-	*ZNF543*	T/C	exon	Y507Y			2.20E-03	0.394
	20	44572085	-	*PCIF1*	G/C	intron				2.52E-03	0.393
	19	8386994	-	*RPS28*	-/C	intron				3.09E-03	-0.335
	7	89861889	-	*STEAP2*	-/T	exon	M475Ifs*51	NA	NA	3.83E-03	-0.328
	8	145059414	rs144026672	*PARP10*	G/A	exon	P264P			5.66E-03	0.345
	15	63433763	-	*LACTB*	-/A	exon	R469Kfs*8	NA	NA	6.56E-03	-0.309
	15	74468404	rs746868961	*ISLR*	A/G	exon	E402G	Tolerated	Benign	6.59E-03	0.313
	17	39190653	-	*KRTAP1-3*	C/A	exon	A141S	Tolerated	Probably damaging	6.73E-03	0.358
	19	37488298	-	*ZNF568*	-/G	exon	E505Gfs*6	NA	NA	6.96E-03	-0.307
	17	40556634	rs3833143	*CAVIN1*	-/GAGCCGAGA	3'UTR				7.10E-03	-0.306
	1	157566015	-	*FCRL4*	-/A	intron				8.19E-03	-0.301
	3	100039903	-	*TBC1D23*	T/G	intron				8.39E-03	0.304
	7	149477851	-	*SSPO*	A/G	intron				9.44E-03	0.320
	11	68772966	-	*MRGPRF*	A/G	exon	F271S	Tolerated	Benign	9.76E-03	0.318

^a^ Based on GRCh37 genome assembly.

^b^ rsID from the NCBI database of genetic variation (dbSNP). "-", this variant is not identified in dbSNP.

^c^ Spearman correlation coefficient had been calculated to estimate positive (resistant) or negative (sensitive) correlation between the VAF and sensitivity to each anticancer drug.

5FU, 5‑fluorouracil; Ref., reference; fs, frameshift; NA, not available.

**Table 2:** Variants associated with chemosensitivity to ACNU (P < 0.01), as identified among 79 xenografts.

Drug	Chr	Position^a^	rsID^b^	Gene	Allele Ref./Variant	Location	Amino acid change	Prediction of functional effect		*P* value	*r*_*s*_^c^
								SIFT	PolyPhen2		
ACNU	3	56650052	rs1553710792	*CCDC66*	-/CTT	exon	T571_S572insF	NA	NA	5.04E-04	0.387
	7	64169017	rs199594424	*ZNF107*	-/GAA	exon	E816delinsGK	NA	NA	7.86E-04	0.375
	12	46760562	rs201547018	*SLC38A2*	C/A	intron				1.17E-03	-0.468
	11	60617832	-	*CCDC86*	-/A	3'UTR				1.81E-03	-0.350
	7	23872045	rs199803936	*STK31*	GA/-	3'UTR				2.69E-03	0.337
	8	96281481	rs142455613	*C8orf37-AS1*	-/GGGGACCTGGC	ncRNA_intron				3.06E-03	0.335
	15	42305854	-	*PLA2G4E*	-/AGG	intron				3.67E-03	-0.327
	9	136135237	rs34229678	*ABO*	AT/GC	exon				3.96E-03	-0.325
	9	15510020	rs148022076	*PSIP1*	-/G	intron				4.00E-03	-0.324
	19	44648728	rs376448556	*ZNF234*	GC/TT	5'UTR				4.81E-03	-0.318
	19	15789257	rs34521056	*CYP4F12*	T/C	intron				5.59E-03	0.313
	10	64967953	rs139722368	*JMJD1C*	AAACCT/-	exon	G939_L940del	NA	NA	6.55E-03	-0.307
	12	5022038	-	*KCNA1*	-/A	3'UTR				7.42E-03	0.303
	8	142231944	rs386730897	*SLC45A4*	GC/AG	intron				7.64E-03	-0.302
	14	75643383	-	*TMED10*	T/G	upstream				7.74E-03	-0.314
	16	81242149	rs386792900	*PKD1L2*	TTT/-	exon	N236del	NA	NA	8.13E-03	-0.300
	2	11348365	rs1553298800	*ROCK2*	-/TAACT	intron				8.33E-03	-0.305
	8	132052227	-	*ADCY8*	G/T	exon	A118D	Tolerated	Benign	8.39E-03	0.315
	22	29655909	-	*RHBDD3*	C/T	3'UTR				8.62E-03	-0.342
	3	50232000	-	*GNAT1*	T/C	exon	S259P	Deleterious	Probably damaging	9.26E-03	-0.336
	2	226447080	rs1292467126	*NYAP2*	A/G	exon	K316R	Tolerated	Benign	9.33E-03	-0.330
	2	119600548	-	*EN1*	G/C	exon	T382S	Deleterious	Benign	9.65E-03	-0.340
	19	50771503	-	*MYH14*	-/G	exon	A931Gfs*46	NA	NA	9.97E-03	-0.296

^a^ Based on GRCh37 genome assembly.

^b^ rsID from the NCBI database of genetic variation (dbSNP). "-", this variant is not identified in dbSNP.

^c^ Spearman correlation coefficient had been calculated to estimate positive (resistant) or negative (sensitive) correlation between the VAF and sensitivity to each anticancer drug.

ACNU, nimustine; Ref., reference; ncRNA, noncoding RNA; del, deletion; ins, insertion; fs, frameshift; NA, not available.

**Table 3:** Variants associated with chemosensitivity to ADR (P < 0.01), as identified among 79 xenografts.

Drug	Chr	Position^a^	rsID^b^	Gene	Allele Ref./Variant	Location	Amino acid change	Prediction of functional effect		*P* value	*r*_*s*_^c^
								SIFT	PolyPhen2		
ADR	8	22960701	-	*LOC254896*, *TNFRSF10C*	-/C	ncRNA_exon, intron				7.15E-05	0.437
	12	56740015	-	*STAT2*	GAA/-	intron				2.59E-03	0.339
	20	7980553	-	*TMX4*	-/A	intron				3.39E-03	-0.330
	22	45914432	-	*FBLN1*	A/T	intron				3.59E-03	0.341
	7	66103436	rs57580125	*KCTD7*	-/AGGA	intron				4.01E-03	-0.326
	6	153312232	rs149540839	*MTRF1L*	-/ATATG	intron				4.14E-03	0.325
	19	10226353	rs71188883	*EIF3G*	TGCC/-	intron				4.41E-03	-0.321
	15	63673951	-	*CA12*	-/G	5'UTR				4.68E-03	0.323
	2	226447080	rs1292467126	*NYAP2*	A/G	exon	K316R	Tolerated	Benign	4.95E-03	-0.355
	3	50155710	-	*SEMA3F-AS1*	A/C	ncRNA_intron				4.98E-03	0.325
	3	148459394	-	*AGTR1*	-/T	exon	I193Dfs*34	NA	NA	5.10E-03	-0.316
	15	78581889	-	*WDR61*	-/T	intron				5.44E-03	0.314
	16	113639	-	*RHBDF1*	G/A	exon	S136S			5.66E-03	0.386
	19	44648728	rs376448556	*ZNF234*	GC/TT	5'UTR				6.52E-03	-0.308
	2	11348365	rs1553298800	*ROCK2*	-/TAACT	intron				6.99E-03	-0.311
	17	47284675	rs3830594	*GNGT2*	-/A	intron				7.79E-03	0.301
	2	74642267	rs768089535	*C2orf81*	-/GCGGAGGGGCGGGTGGCGCCGCCC	exon	A251delins GAAPPAPPP	NA	NA	7.89E-03	0.301
	6	24450169	rs5874981	*GPLD1*	-/CCT	intron				8.61E-03	0.297
	20	238437	-	*DEFB132*	GGTCTT/-	exon	V7_L8del	NA	NA	9.50E-03	-0.296

^a^ Based on GRCh37 genome assembly.

^b^ rsID from the NCBI database of genetic variation (dbSNP). "-", this variant is not identified in dbSNP.

^c^ Spearman correlation coefficient had been calculated to estimate positive (resistant) or negative (sensitive) correlation between the VAF and sensitivity to each anticancer drug.

ADR, adriamycin; Ref., reference; ncRNA, noncoding RNA; del, deletion; ins, insertion; fs, frameshift; NA, not available.

**Table 4:** Variants associated with chemosensitivity to CPM (P < 0.01), as identified among 79 xenografts.

Drug	Chr	Position^a^	rsID^b^	Gene	Allele Ref./Variant	Location	Amino acid change	Prediction of functional effect		*P* value	*r*_*s*_^c^
								SIFT	PolyPhen2		
CPM	16	89656247	-	*CPNE7*	-/C	intron				9.17E-05	0.426
	10	79588623	rs58805712	*DLG5*	CCAGCCT/-	intron				1.14E-04	0.420
	18	64178922	rs1222906114	*CDH19*	C/A	exon	V487L	Tolerated	Benign	6.42E-04	-0.458
	22	20096315	rs57382195	*DGCR8*	-/CGCCTACCTTGCC AGACCCTGGGCA	intron				1.09E-03	0.361
	7	23854839	rs5882915	*STK31*	-/A	intron				2.17E-03	0.340
	16	775954	rs373524109	*CCDC78*	CC/AA	intron				2.21E-03	0.339
	1	228529430	rs71180792	*OBSCN*	-/GACGGCTCAGCCAG CCTGTGGCATGG	intron				2.30E-03	-0.356
	6	24450169	rs5874981	*GPLD1*	-/CCT	intron				2.43E-03	0.337
	5	112406786	rs11283943	*MCC*	-/CCTCGCGCTGTCTT	intron				2.79E-03	-0.332
	3	197428754	-	*RUBCN*	C/A	intron				3.57E-03	-0.417
	5	56226601	-	*MIER3*	-/A	intron				3.74E-03	0.323
	7	123190494	rs4147636	*NDUFA5*	-/CTGGATACCACAAATC	intron				3.97E-03	-0.335
	10	73571582	rs59718926	*CDH23*	-/CT	intron				3.97E-03	-0.321
	2	226447080	rs1292467126	*NYAP2*	A/G	exon	K316R	Tolerated	Benign	3.98E-03	-0.361
	17	79687211	-	*SLC25A10*	G/T	exon	W295C	NA	NA	4.04E-03	-0.382
	17	46691588	rs11267100	*HOXB8*	-/GGCCCCCTGCCC	intron				4.92E-03	-0.315
	9	136507571	-	*DBH*	G/T	exon	R243R			5.38E-03	-0.404
	1	1510022	-	*SSU72*	G/T	5'UTR				5.45E-03	-0.341
	1	38334317	rs386630429	*INPP5B*	TC/AA	intron				5.47E-03	0.310
	16	48244838	rs35914140	*ABCC11*	GC/AA	intron				5.77E-03	0.308
	19	49134387	-	*DBP*	-/C	intron				5.87E-03	0.307
	1	248367014	-	*OR2M3*	TG/CA	exon	A216T	NA	NA	5.89E-03	-0.307
	1	206681225	-	*RASSF5*	C/A	exon	A97E	NA	Possibly damaging	7.10E-03	-0.339
	4	170038575	rs373660112	*SH3RF1*	AA/TT	intron				7.32E-03	0.300
	22	45914431	rs11421543	*FBLN1*	-/T	intron				7.92E-03	0.297
	3	186338529	-	*AHSG*	C/T	exon	P305L	Tolerated	Benign	7.98E-03	0.315
	11	96123735	rs3842515	*JRKL*	-/G	5'UTR				9.24E-03	-0.291
	2	27884266	-	*SUPT7L*	T/G	5'UTR				9.68E-03	0.297
	5	111500819	-	*EPB41L4A*	-/AAAT	intron				9.79E-03	-0.289

^a^ Based on GRCh37 genome assembly.

^b^ rsID from the NCBI database of genetic variation (dbSNP). "-", this variant is not identified in dbSNP.

^c^ Spearman correlation coefficient had been calculated to estimate positive (resistant) or negative (sensitive) correlation between the VAF and sensitivity to each anticancer drug.

CPM, cyclophosphamide; Ref., reference; NA, not available.

**Table 5:** Variants associated with chemosensitivity to DDP (P < 0.01), as identified among 79 xenografts.

Drug	Chr	Position^a^	rsID^b^	Gene	Allele Ref./Variant	Location	Amino acid change	Prediction of functional effect		*P* value	*r*_*s*_^c^
								SIFT	PolyPhen2		
DDP	3	50155710	-	*SEMA3F-AS1*	A/C	ncRNA_intron				6.66E-04	0.389
	21	45843709	rs765207853	*TRPM2-AS*	AGG/-	ncRNA_intron				1.44E-03	-0.357
	19	40541037	-	*ZNF780B*	-/T	exon	S577Kfs*9	NA	NA	1.58E-03	0.354
	7	64169017	rs199594424	*ZNF107*	-/GAA	exon	E816delinsGK	NA	NA	1.73E-03	0.351
	7	23854839	rs5882915	*STK31*	-/A	intron				1.77E-03	0.351
	7	123190494	rs4147636	*NDUFA5*	-/CTGGATACC ACAAATC	intron				3.23E-03	-0.347
	17	7225146	-	*NEURL4*	-/G	intron				4.74E-03	-0.319
	19	2980268	-	*TLE6*	-/A	intron				5.03E-03	-0.317
	3	47453783	-	*PTPN23*	G/T	exon	G1271C	Deleterious	Probably damaging	5.55E-03	0.324
	15	78581889	-	*WDR61*	-/T	intron				5.68E-03	0.312
	15	73994678	rs1271868805	*CD276*	T/C	exon	P54P			6.42E-03	-0.351
	5	147695284	rs3217238	*LOC102546294*	-/TCA	ncRNA_intron				6.63E-03	-0.307
	6	350941	rs11408655	*DUSP22*	-/A	3'UTR				6.69E-03	0.307
	13	50092133	-	*PHF11*, *SETDB2-PHF11*	GTA/-	intron, intron				7.21E-03	0.319
	6	36759740	-	*CPNE5*	-/A	intron				7.38E-03	-0.303
	8	124749609	-	*ANXA13*	-/T	5'UTR				7.70E-03	-0.302
	19	21300592	-	*ZNF714*	C/A	exon	G374G			7.89E-03	-0.355
	22	20784908	rs35574298	*SCARF2*	TT/GA	intron				8.26E-03	0.299
	6	28331127	rs371085669	*ZKSCAN3*	AA/GC	exon	K52A	NA	NA	9.56E-03	0.294
	1	85787116	rs1172711726	*DDAH1*	-/C	3'UTR				9.92E-03	-0.292
	7	111846724	rs773775063	*ZNF277*	C/A	5'UTR				9.97E-03	0.372

^a^ Based on GRCh37 genome assembly.

^b^ rsID from the NCBI database of genetic variation (dbSNP). "-", this variant is not identified in dbSNP.

^c^ Spearman correlation coefficient had been calculated to estimate positive (resistant) or negative (sensitive) correlation between the VAF and sensitivity to each anticancer drug.

DDP, cisplatin; Ref., reference; ncRNA, noncoding RNA; del, deletion; ins, insertion; fs, frameshift; NA, not available.

**Table 6:** Variants associated with chemosensitivity to MMC (P < 0.01), as identified among 79 xenografts.

Drug	Chr	Position^a^	rsID^b^	Gene	Allele Ref./Variant	Location	Amino acid change	Prediction of functional effect		*P* value	*r*_*s*_^c^
								SIFT	PolyPhen2		
MMC	2	242078024	-	*PASK*	C/A	intron				2.05E-03	0.397
	8	68993013	rs368406603	*PREX2*	AT/GC	exon	F605F			3.11E-03	-0.329
	21	47910655	-	*DIP2A*	-/G	intron				3.21E-03	0.328
	9	71098986	rs377702519	*PGM5*	CA/TG	intron				4.84E-03	0.314
	12	58335540	-	*ATP23*	A/T	exon	Q19L	Tolerated	Benign	6.58E-03	0.322
	1	22852713	-	*ZBTB40*	-/C	exon	V1071Gfs*34	NA	NA	7.09E-03	0.301
	3	56593542	-	*CCDC66*	-/A	intron				7.59E-03	-0.298
	18	47320561	rs35615995	*ACAA2*	-/TAAA	intron				7.93E-03	-0.299
	5	79368050	-	*CTD-2201I18*.*1*	GAA/-	ncRNA_intron				8.47E-03	-0.294
	6	52138176	-	*MCM3*	-/A	intron				8.65E-03	0.294
	15	78581889	-	*WDR61*	-/T	intron				8.71E-03	0.293

^a^ Based on GRCh37 genome assembly.

^b^ rsID from the NCBI database of genetic variation (dbSNP). "-", this variant is not identified in dbSNP.

^c^ Spearman correlation coefficient had been calculated to estimate positive (resistant) or negative (sensitive) correlation between the VAF and sensitivity to each anticancer drug.

MMC, mitomycin C; Ref., reference; ncRNA, noncoding RNA; fs, frameshift; NA, not available.

**Table 7:** Variants associated with chemosensitivity to MTX (P < 0.01), as identified among 79 xenografts.

Drug	Chr	Position^a^	rsID^b^	Gene	Allele Ref./Variant	Location	Amino acid change	Prediction of functional effect		*P* value	*r*_*s*_^c^
								SIFT	PolyPhen2		
MTX	5	145508636	rs1554127194	*LARS*	TA/CC	exon	N846D	NA	NA	2.11E-03	-0.366
	20	16316676	-	*KIF16B*	A/C	intron				2.81E-03	0.365
	19	39961019	-	*SUPT5H*	-/C	exon	K508Qfs*22	NA	NA	3.22E-03	0.368
	3	183013150	-	*MCF2L2*	G/T	exon	A538D	Deleterious	Probably damaging	3.36E-03	-0.399
	5	131705587	rs71590771	*MIR3936HG*	CG/TA	ncRNA_exon				3.46E-03	0.350
	3	101484334	-	*CEP97*	-/A	exon	Q789Afs*5	NA	NA	3.53E-03	0.349
	7	12391269	rs11454536	*VWDE*	-/A	exon	K1158fs*0	NA	NA	3.82E-03	0.346
	2	189916175	-	*COL5A2*	T/G	exon	E934D	Deleterious	Benign	3.99E-03	0.400
	15	22960698	-	*CYFIP1*	-/G	intron				5.35E-03	-0.334
	22	26709896	-	*SEZ6L*	-/A	intron				6.00E-03	-0.330
	20	43108927	rs1356710132	*TTPAL*	G/A	exon	L96L			6.25E-03	0.364
	11	47201752	-	*PACSIN3*	G/T	exon	A196A			6.25E-03	-0.382
	17	21215643	rs73302034	*MAP2K3*	A/G	intron				7.11E-03	0.324
	17	7721209	-	*DNAH2*	T/C	intron				7.42E-03	-0.327
	1	146763088	-	*CHD1L*, *NBPF19*	-/A	intron, intron				7.47E-03	0.322
	2	172216968	rs112550880	*METTL8*	-/C	exon	E22Gfs*13	NA	NA	7.85E-03	-0.320
	17	8046598	-	*PER1*	G/T	exon	P1020T	Tolerated	Possibly damaging	8.34E-03	-0.373
	2	232087473	-	*ARMC9*	-/G	exon	I180Dfs*8	NA	NA	8.48E-03	0.317
	7	48146989	-	*UPP1*	C/T	exon	S89S			8.55E-03	-0.392
	7	150883555	-	*ASB10*	C/A	exon	A170S	Tolerated	Benign	8.93E-03	-0.363
	14	97313771	rs143447703	*VRK1*	-/A	intron				9.12E-03	0.314
	17	47284675	rs3830594	*GNGT2*	-/A	intron				9.65E-03	0.312
	7	111846724	rs773775063	*ZNF277*	C/A	5'UTR				9.87E-03	0.398
	3	196296147	rs1560314855	*FBXO45*	T/G	exon	C98G	Tolerated	Benign	9.91E-03	-0.323

^a^ Based on GRCh37 genome assembly.

^b^ rsID from the NCBI database of genetic variation (dbSNP). "-", this variant is not identified in dbSNP.

^c^ Spearman correlation coefficient had been calculated to estimate positive (resistant) or negative (sensitive) correlation between the VAF and sensitivity to each anticancer drug.

MTX, methotrexate; Ref., reference; ncRNA, noncoding RNA; fs, frameshift; NA, not available.

**Table 8:** Variants associated with chemosensitivity to VCR (P < 0.01), as identified among 79 xenografts.

Drug	Chr	Position^a^	rsID^b^	Gene	Allele Ref./Variant	Location	Amino acid change	Prediction of functional effect		*P* value	*r*_*s*_^c^
								SIFT	PolyPhen2		
VCR	3	53220765	rs3830265	*PRKCD*	TCAGAGCC/-	intron				2.38E-03	-0.343
	4	151187012	rs150278643	*LRBA*	-/GAGAT	intron				2.81E-03	0.338
	22	45182326	rs67401095	*ARHGAP8*, *PRR5-ARHGAP8*	CTT/-	intron, intron				3.08E-03	-0.335
	10	73115941	rs34040486	*SLC29A3*	TG/CA	exon	V239I	NA	NA	3.12E-03	-0.335
	1	248367014	-	*OR2M3*	TG/CA	exon	A216T	NA	NA	3.47E-03	-0.331
	14	77751922	-	*POMT2*	-/T	exon	F463Ifs*77	NA	NA	3.87E-03	0.328
	4	184619035	rs200831837	*TRAPPC11*	C/T	intron				4.46E-03	0.323
	18	40695532	-	*RIT2*	T/C	5'UTR				5.29E-03	-0.392
	19	21300592	-	*ZNF714*	C/A	exon	G374G			5.34E-03	-0.374
	3	24006476	-	*NR1D2*	-/A	exon	L311Ifs*2	NA	NA	5.52E-03	-0.315
	15	43028509	-	*CDAN1*	-/C	exon	T189Yfs*45	NA	NA	6.03E-03	0.312
	19	21606610	-	*ZNF493*	C/A	exon	G255G			6.22E-03	-0.361
	2	226447080	rs1292467126	*NYAP2*	A/G	exon	K316R	Tolerated	Benign	6.22E-03	-0.347
	19	21240167	-	*ZNF430*	C/A	exon	G350G			6.31E-03	-0.349
	3	124646709	rs869290005	*MUC13*	-/AAG	exon	T60_P61insL	NA	NA	6.35E-03	-0.310
	19	36355594	-	*KIRREL2*	-/G	exon	K541Efs*50	NA	NA	6.47E-03	0.310
	16	22161135	-	*VWA3A*	-/C	exon	F1005Lfs*16	NA	NA	7.42E-03	-0.305
	20	948071	-	*RSPO4*	T/C	intron				8.00E-03	-0.357
	12	75816816	rs59277111	*GLIPR1L2*	-/CAA	exon	D239_K240insQ	NA	NA	8.66E-03	-0.299
	17	47888851	-	*KAT7*	-/G	exon	Q88Tfs*4	NA	NA	8.88E-03	-0.298
	10	78084100	rs372941859	*LRMDA*	GG/CC	intron				9.29E-03	-0.297
	19	20002842	-	*ZNF253*	C/A	exon	G186G			9.46E-03	-0.347
	3	50232000	-	*GNAT1*	T/C	exon	S259P	Deleterious	Probably damaging	9.58E-03	-0.337
	8	74335015	rs60338415	*STAU2-AS1*	-/AGAAAGAC	ncRNA_intron				9.77E-03	0.297
	20	57430029	-	*GNAS*	C/A	exon	P508T	Tolerated	Benign	9.94E-03	-0.325
	1	67423998	rs142198730	*MIER1*	-/TTCTC	intron				9.95E-03	-0.302

^a^ Based on GRCh37 genome assembly.

^b^ rsID from the NCBI database of genetic variation (dbSNP). "-", this variant is not identified in dbSNP.

^c^ Spearman correlation coefficient had been calculated to estimate positive (resistant) or negative (sensitive) correlation between the VAF and sensitivity to each anticancer drug.

VCR, vincristine; Ref., reference; ncRNA, noncoding RNA; ins, insertion; fs, frameshift; NA, not available.

**Table 9:** Variants associated with chemosensitivity to VLB (P < 0.01), as identified among 79 xenografts.

Drug	Chr	Position^a^	rsID^b^	Gene	Allele Ref./Variant	Location	Amino acid change	Prediction of functional effect		*P* value	*r*_*s*_^c^
								SIFT	PolyPhen2		
VLB	10	45869697	-	*ALOX5*	-/C	5'UTR				1.65E-03	0.355
	1	169679473	rs4987281	*SELL*	-/CT	intron				1.96E-03	0.350
	3	169540395	-	*LRRIQ4*	-/C	exon	C231Vfs*3	NA	NA	2.82E-03	-0.340
	9	132652688	-	*FNBP1*	-/GAC	3'UTR				4.06E-03	0.326
	4	184619035	rs200831837	*TRAPPC11*	C/T	intron				4.16E-03	0.325
	8	96281481	rs142455613	*C8orf37-AS1*	-/GGGGACCTGGC	ncRNA_intron				4.21E-03	0.327
	4	6293234	-	*WFS1*	-/G	intron				5.35E-03	0.316
	7	1528998	-	*INTS1*	AT/CA	exon	M767W	NA	NA	6.08E-03	0.312
	22	50659594	-	*TUBGCP6*	TC/CT	exon	E1065R	NA	NA	6.52E-03	0.309
	7	111846724	rs773775063	*ZNF277*	C/A	5'UTR				6.54E-03	0.395
	6	74123314	rs35252896	*DDX43*	-/GCT	intron				8.14E-03	-0.301
	7	12391269	rs11454536	*VWDE*	-/A	exon	K1158fs*0	NA	NA	8.80E-03	0.299
	13	24869045	-	*SPATA13*	-/C	intron				8.97E-03	0.298
	19	21300592	-	*ZNF714*	C/A	exon	G374G			9.32E-03	-0.351
	7	111846719	-	*ZNF277*	C/A	5'UTR				9.88E-03	0.369
	2	160075887	rs3214491	*TANC1*	-/C	intron				9.90E-03	-0.294

^a^ Based on GRCh37 genome assembly.

^b^ rsID from the NCBI database of genetic variation (dbSNP). "-", this variant is not identified in dbSNP.

^c^ Spearman correlation coefficient had been calculated to estimate positive (resistant) or negative (sensitive) correlation between the VAF and sensitivity to each anticancer drug.

VLB, vinblastine; Ref., reference; ncRNA, noncoding RNA; fs, frameshift; NA, not available.

For the other eight drugs, the variants most strongly associated with chemosensitivity were as follows (Tables 1–9): NIMA-related kinase 1 (*NEK1*) showed strong associations with 5FU treatment (*P* = 1.76 × 10^−3^, *r*_*s*_ = -0.355, Table 1); coiled-coil domain containing 66 (*CCDC66*) with ACNU (*P* = 5.04 × 10^−4^, *r*_*s*_ = 0.387, Table 2); copine 7 (*CPNE7*) with CPM (*P* = 9.17 × 10^−5^, *r*_*s*_ = 0.426, Table 4); SEMA3F antisense RNA 1 (*SEMA3F-AS1*) with DDP (*P* = 6.66 × 10^−4^, *r*_*s*_ = 0.389, Table 5); PAS domain-containing serine/threonine kinase (*PASK*) with MMC (*P* = 2.05 × 10^−3^, *r*_*s*_ = 0.397, Table 6); leucyl-tRNA synthetase 1 (*LARS*) with MTX (*P* = 2.11 × 10^−3^, *r*_*s*_ = -0.366, Table 7); protein kinase C delta (*PRKCD*) with VCR (*P* = 2.38 × 10^−3^, *r*_*s*_ = -0.343, Table 8); and arachidonate 5-lipoxygenase (*ALOX5*) with VLB (*P* = 1.65 ×10^−3^, *r*_*s*_ = 0.355, Table 9).

### Genetic variants associated with multi-drug sensitivity

**Fig 2:**
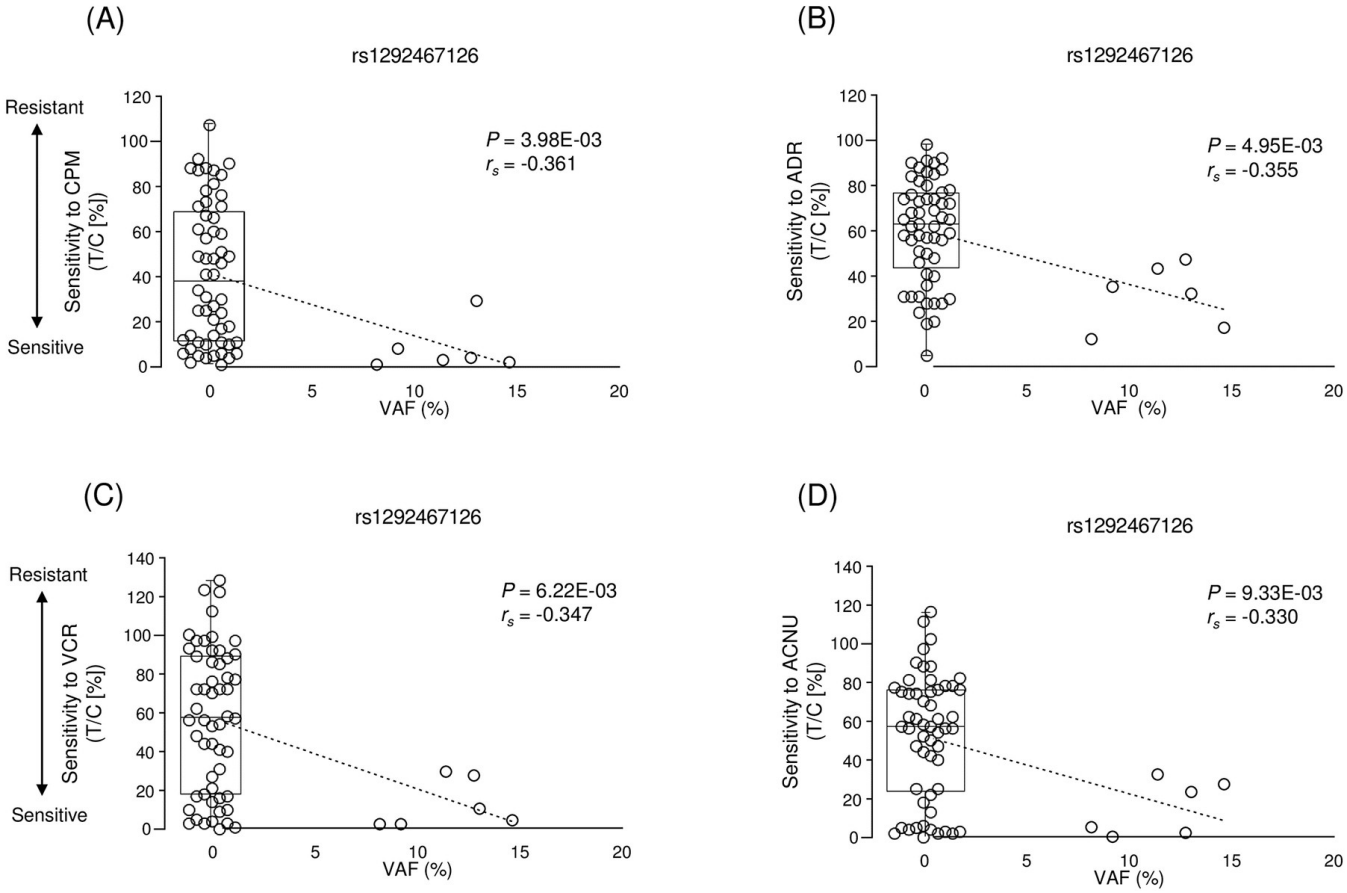
Correlation between variant rs1292467126 and chemosensitivity to CPM (A), ADR (B), VCR (C), and ACNU (D). Chemosensitivity to each drug is represented by relative tumor volume of treated mice (T) with respect to that of the control mice (C). rs1292467126 was commonly associated with increased sensitivity to four of the nine tested anticancer drugs.

There were 162 variants possibly associated with chemosensitivity to more than one of the nine anticancer drugs (*P* < 0.01, Tables 1–9). rs1292467126 (chr2:g.226447080 A>G) in exon 4 of neuronal tyrosine-phosphorylated phosphoinositide-3-kinase adaptor 2 (*NYAP2*) was the most commonly associated variant, with chemosensitivity to four anti-cancer drugs: CPM (*P* = 3.98 × 10^−3^, *r*_*s*_ = -0.361; Table 4), ADR (*P* = 4.95 × 10^−3^, *r*_*s*_ = -0.355; Table 3), VCR (*P* = 6.22 × 10^−3^, *r*_*s*_ = -0.347; Table 8), and ACNU (*P* = 9.33 × 10^−3^, *r*_*s*_ = -0.330; Table 2). Xenografts with higher VAFs of rs1292467126 had better responses to the four drugs, as shown in Table 10 and Fig 2. Furthermore, three variants were associated with three drugs and 13 variants with two drugs (Table 10). For example, rs773775063 (chr7:g.111846724 C>A) in the 5’UTR of zinc finger protein 277 (*ZNF277*) was associated with resistance to VLB (*P* = 6.54 × 10^−3^, *r*_*s*_ = 0.395), MTX (*P* = 9.87 × 10^−3^, *r*_*s*_ = 0.398), and DDP (*P* = 9.97 × 10^−3^, *r*_*s*_ = 0.372) (Table 10).

**Table 10:** Variants commonly associated with chemosensitivity to two or more anticancer drugs (P < 0.01).

Chr	Position^a^	rsID^b^	Gene	Allele Ref./Variant	Location	Amino acid change	Prediction of functional effect		*P* value	*r*_*s*_^c^	Drug
							SIFT	PolyPhen2			
2	226447080	rs1292467126	*NYAP2*	A/G	exon	K316R	Tolerated	Benign	3.98E-03	-0.361	CPM
									4.95E-03	-0.355	ADR
									6.22E-03	-0.347	VCR
									9.33E-03	-0.330	ACNU
19	21300592	-	*ZNF714*	C/A	exon	G374G			5.34E-03	-0.374	VCR
									7.89E-03	-0.355	DDP
									9.32E-03	-0.351	VLB
15	78581889	-	*WDR61*	-/T	intron				5.44E-03	0.314	ADR
									5.68E-03	0.312	DDP
									8.71E-03	0.293	MMC
7	111846724	rs773775063	*ZNF277*	C/A	5'UTR				6.54E-03	0.395	VLB
									9.87E-03	0.398	MTX
									9.97E-03	0.372	DDP
3	50155710	-	*SEMA3F-AS1*	A/C	ncRNA_intron				6.66E-04	0.389	DDP
									4.98E-03	0.325	ADR
7	64169017	rs199594424	*ZNF107*	-/GAA	exon	E816delinsGK	NA	NA	7.86E-04	0.375	ACNU
									1.73E-03	0.351	DDP
7	23854839	rs5882915	*STK31*	-/A	intron				1.77E-03	0.351	DDP
									2.17E-03	0.340	CPM
6	24450169	rs5874981	*GPLD1*	-/CCT	intron				2.43E-03	0.337	CPM
									8.61E-03	0.297	ADR
8	96281481	rs142455613	*C8orf37-AS1*	-/GGGGACCTGGC	ncRNA_intron				3.06E-03	0.335	ACNU
									4.21E-03	0.327	VLB
7	123190494	rs4147636	*NDUFA5*	-/CTGGATACCACAAATC	intron				3.23E-03	-0.347	DDP
									3.97E-03	-0.335	CPM
1	248367014	-	*OR2M3*	TG/CA	exon	A216T	NA	NA	3.47E-03	-0.331	VCR
									5.89E-03	-0.307	CPM
7	12391269	rs11454536	*VWDE*	-/A	exon	K1158fs*0	NA	NA	3.82E-03	0.346	MTX
									8.80E-03	0.299	VLB
4	184619035	rs200831837	*TRAPPC11*	C/T	intron				4.16E-03	0.325	VLB
									4.46E-03	0.323	VCR
19	44648728	rs376448556	*ZNF234*	GC/TT	5'UTR				4.81E-03	-0.318	ACNU
									6.52E-03	-0.308	ADR
2	11348365	rs1553298800	*ROCK2*	-/TAACT	intron				6.99E-03	-0.311	ADR
									8.33E-03	-0.305	ACNU
17	47284675	rs3830594	*GNGT2*	-/A	intron				7.79E-03	0.301	ADR
									9.65E-03	0.312	MTX
3	50232000	-	*GNAT1*	T/C	exon	S259P	Deleterious	Probably damaging	9.26E-03	-0.336	ACNU
									9.58E-03	-0.337	VCR

^a^ Based on GRCh37 genome assembly.

^b^ rsID from the NCBI database of genetic variation (dbSNP). "-", this variant is not identified in dbSNP.

^c^ Spearman correlation coefficient had been calculated to estimate positive (resistant) or negative (sensitive) correlation between the VAF and sensitivity to each anticancer drug.

ACNU, nimustine; ADR, adriamycin; CPM, cyclophosphamide; DDP, cisplatin; MMC, mitomycin C; MTX, methotrexate; VCR, vincristine; VLB, vinblastine; ncRNA, noncoding RNA; del, deletion; ins, insertion; fs, frameshift; NA, not available.

### Subgroup analysis

We further performed a subgroup analysis based on cancer type to identify tissue-specific chemosensitivity-related variants. Subgroups of breast and gastric cancers were analyzed because more than 10 xenografts of these cancer types were available. In breast and gastric cancer xenografts, 78 and 67 variants, respectively, were possibly associated with chemosensitivity to one or more drugs, with *P* values < 0.01 (Tables 11 and 12). rs386792906 (chr16:g.81253642 AG>TC) in polycystin 1 like 2 (*PKD1L2*), which was associated with resistance to MTX, showed the strongest association of the nine tested anti-cancer drugs among the breast cancer subgroup (*P* = 1.52 × 10^−5^, *r*_*s*_ = 0.991, Table 11). rs73302038 (chr17:g.21215682 G>A) in mitogen-activated protein kinase kinase 3 (*MAP2K3*), which was associated with resistance to VCR, showed the strongest association for the gastric cancer subgroup (*P* = 8.32 × 10^−6^, *r*_*s*_ = 0.935, Table 12). However, of the variants with *P* < 0.01 in the subgroup analyses, only three (chr15:g. 22960698 insG in MTX, rs59277111 in VCR, and rs3217238 in DDP) were significant at *P* < 0.01 in the whole-group analysis (Tables 11 and 12).

**Table 11:** Variants associated with chemosensitivity to each drug (P < 0.01) in breast cancer xenografts.

Drug	Chr	Position^a^	rsID^b^	Gene	Allele Ref./Variant	Location	Amino acid change	Prediction of functional effect		Breast cancer			All xenografts		
								SIFT	PolyPhen2	N	*P* value	*r*_*s*_^c^	N	*P* value	*r*_*s*_^c^
5FU	1	236978993	rs35668201	*MTR*	-/TCTG	intron				10	5.42E-04	0.891	76	4.69E-01	0.084
	11	76901624	rs35298297	*MYO7A*	-/GCTGGGGC CTGGAGC	intron				9	7.34E-04	-0.907	70	6.96E-01	-0.048
	15	34634138	rs386782889	*NOP10*	GT/AC	3'UTR				10	4.16E-03	0.814	76	2.43E-02	0.258
	4	84230617	-	*HPSE*	-/C	exon	E250Gfs*6	NA	NA	10	5.69E-03	-0.798	76	2.47E-02	-0.257
	1	234458667	-	*SLC35F3*	-/A	intron				10	5.69E-03	0.798	76	5.31E-02	0.223
	21	47985555	rs74854320	*DIP2A*	-/G	intron				10	6.38E-03	0.791	76	4.83E-02	0.227
	21	40883673	-	*SH3BGR*, *WRB-SH3BGR*	-/GAA	exon, exon	E200delinsGK, E265delinsGK	NA	NA	10	6.51E-03	-0.790	76	2.15E-01	-0.144
	20	13763897	rs28372964	*ESF1*	-/ATTA	intron				10	7.55E-03	-0.782	75	8.83E-01	-0.017
	1	228529430	rs71180792	*OBSCN*	-/GACGGCTCAGCCA GCCTGTGGCATGG	intron				8	8.35E-03	-0.844	68	9.76E-01	0.004
	3	156175167	rs34680920	*KCNAB1*	GG/AA	intron				10	8.75E-03	0.773	76	3.18E-01	0.116
	10	71648214	rs74503792	*COL13A1*	-/A	intron				10	9.47E-03	-0.768	76	2.72E-01	-0.128
ACNU	11	96123735	rs3842515	*JRKL*	-/G	5'UTR				11	6.67E-04	-0.861	77	5.72E-01	-0.065
	6	33659469	-	*ITPR3*	-/G	exon	L2436Afs*4	NA	NA	11	1.08E-03	-0.844	77	9.80E-01	-0.003
	6	152461050	-	*SYNE1*	GTTT/-	intron				11	1.94E-03	-0.821	77	2.58E-01	-0.13
	6	30558478	-	*ABCF1*	-/A	exon	X808delinsX	NA	NA	11	2.00E-03	-0.820	77	6.94E-01	0.046
	10	78084100	rs372941859	*LRMDA*	GG/CC	intron				11	4.91E-03	0.777	77	5.08E-01	-0.077
	19	2980268	-	*TLE6*	-/A	intron				11	5.99E-03	-0.766	77	4.99E-02	-0.224
	6	42853640	-	*RPL7L1*	-/TCC	intron				11	6.86E-03	0.758	77	4.60E-01	0.085
	4	69964337	rs386675647	*UGT2B7*	AT/TC	exon	Y268H	NA	NA	11	8.29E-03	0.747	77	8.67E-01	0.019
	4	6594943	-	*MAN2B2*	-/C	exon	Q243Pfs*9	NA	NA	11	8.57E-03	-0.745	77	7.38E-01	0.039
ADR	14	69791440	-	*GALNT16*	C/A	exon	P123T	Deleterious	Probably damaging	10	6.43E-04	0.886	60	9.04E-01	-0.016
	11	56468448	rs1554964167	*OR9G1*, *OR9G9*	AA/GT	exon, exon	I196F, I196F	NA	NA	11	2.88E-03	-0.804	77	4.33E-02	-0.231
	12	6886294	-	*LAG3*	A/G	intron				8	3.18E-03	0.889	51	3.70E-02	0.293
	16	4254408	rs1555454446	*SRL*	-/AGATACAGCCC CGGCCTCCA	intron				11	3.63E-03	-0.792	76	7.37E-01	0.039
	14	75745752	-	*FOS*	G/C	exon	A23P	Tolerated	Probably damaging	7	4.80E-03	0.907	67	5.00E-01	0.084
	7	100361392	rs3215395	*ZAN*	-/C	intron				11	5.83E-03	0.767	77	4.18E-01	0.094
	5	1093610	rs56276350	*SLC12A7*	-/GGGCGGGGACT	intron				10	5.93E-03	0.795	73	5.48E-01	0.071
	10	73571582	rs59718926	*CDH23*	-/CT	intron				11	7.44E-03	-0.753	77	5.28E-01	-0.073
	16	138773	rs57321480	*NPRL3*	-/G	exon				11	7.82E-03	0.750	77	9.47E-01	0.008
	22	50927448	rs55651311	*MIOX*	-/GTCCCTCCT	intron				10	9.83E-03	-0.766	71	6.24E-01	-0.059
CPM	6	42853640	-	*RPL7L1*	-/TCC	intron				12	3.32E-03	0.771	79	1.56E-01	0.161
	10	78084100	rs372941859	*LRMDA*	GG/CC	intron				12	3.43E-03	0.769	79	8.48E-01	0.022
	3	49148887	-	*USP19*	-/C	intron				12	4.10E-03	0.760	79	2.86E-01	0.122
	7	100229467	-	*TFR2*	G/T	exon	A185A			8	5.15E-03	0.868	42	1.03E-01	0.255
	1	234458667	-	*SLC35F3*	-/A	intron				12	8.52E-03	0.718	79	4.80E-02	0.223
	1	164781111	rs869176116	*PBX1*	-/ATATAAG	intron				12	9.83E-03	0.709	75	3.10E-02	0.249
	11	66333595	-	*CTSF*	-/T	exon	E256Rfs*13	NA	NA	12	9.98E-03	-0.708	79	1.85E-02	-0.264
DDP	5	137682568	-	*FAM53C*	G/T	exon	G367C	Deleterious	Benign	6	7.14E-04	0.978	46	7.39E-01	0.051
	6	42853640	-	*RPL7L1*	-/TCC	intron				11	1.14E-03	0.842	77	2.29E-01	0.139
	10	78084100	rs372941859	*LRMDA*	GG/CC	intron				11	1.32E-03	0.837	77	7.23E-01	0.041
	4	69964337	rs386675647	*UGT2B7*	AT/TC	exon	Y268H	NA	NA	11	3.68E-03	0.792	77	3.46E-01	0.109
	18	67863852	rs386388096	*RTTN*	-/TCC	exon	G242_D243insE	NA	NA	11	5.30E-03	-0.773	77	9.17E-01	-0.012
	2	128878011	-	*UGGT1*	-/A	exon	V320Gfs*20	NA	NA	11	5.71E-03	0.769	77	3.69E-01	0.104
	17	15343525	rs66754946	*CDRT4*	-/CTT	exon	E9_V10insK	NA	NA	11	6.05E-03	-0.765	77	2.70E-01	-0.127
	10	134012502	-	*DPYSL4*	CCGAGGGG/-	intron				11	7.02E-03	0.757	77	9.36E-01	0.009
	6	8073625	-	*EEF1E1-BLOC1S5*	AGAGTAGTTT/-	ncRNA_intron				11	7.65E-03	-0.752	77	2.92E-01	0.122
	1	228469903	rs386640014	*OBSCN*	AG/TT	exon	R2823L	NA	NA	11	8.60E-03	0.744	77	8.38E-01	0.024
	1	228476366	rs386640016	*OBSCN*	GA/TT	exon	E3372_S3373 delinsDC	NA	NA	11	8.72E-03	0.744	77	3.83E-01	0.101
	3	172473062	-	*ECT2*	-/AT	intron				11	9.22E-03	0.740	77	5.68E-01	0.066
	11	96123735	rs3842515	*JRKL*	-/G	5'UTR				11	9.43E-03	-0.739	77	5.06E-01	0.077
MMC	12	113444417	-	*OAS2*	-/C	intron				12	2.85E-04	-0.864	79	3.18E-02	-0.242
	22	36587848	rs869115251	*APOL4*	-/CT	exon				12	3.66E-04	0.857	79	2.82E-01	0.123
	16	772841	-	*CCDC78*	-/C	intron				12	2.49E-03	-0.785	79	8.15E-01	0.027
	5	26906240	-	*CDH9*	T/G	intron				10	4.95E-03	-0.805	76	2.91E-01	-0.123
	22	38318262	-	*MICALL1*	C/A	exon	R285R			9	6.15E-03	-0.825	68	9.52E-01	-0.008
	6	44274143	-	*AARS2*	GAA/-	intron				12	6.99E-03	0.730	79	1.56E-01	0.161
	1	203816588	-	*ZC3H11A*	T/A	exon	I440N	Tolerated	Benign	7	9.74E-03	0.876	53	8.81E-02	-0.237
MTX	16	81253642	rs386792906	*PKD1L2*	AG/TC	intron				7	1.52E-05	0.991	68	8.70E-01	0.02
	11	5411579	rs369353765	*OR51M1*	GT/AC	exon	F318L	NA	NA	7	4.97E-04	0.963	68	4.54E-01	0.092
	21	45843709	rs765207853	*TRPM2-AS*	AGG/-	ncRNA_intron				7	8.67E-04	0.954	68	1.26E-02	0.301
	6	80513567	-	*LINC01621*	-/TCTCTGATA TGCCATCC	ncRNA_exon				7	1.30E-03	-0.945	68	4.67E-01	-0.09
	22	20784908	rs35574298	*SCARF2*	TT/GA	intron				7	2.06E-03	-0.934	68	1.43E-01	-0.18
	17	7123256	-	*ACADVL*, *DLG4*	-/GGGCGTGC AGGACGC	5'UTR, 5'UTR				7	3.92E-03	0.915	68	5.76E-01	-0.069
	15	22960698	-	*CYFIP1*	-/G	intron				7	5.13E-03	-0.905	68	5.35E-03	-0.334
	6	30314566	rs35287137	*RPP21*, *TRIM39-RPP21*	TC/GA	exon, exon	Q157K, Q498K	NA	NA	7	6.53E-03	0.895	68	5.92E-01	-0.066
	9	88631383	rs368374310	*NAA35*	-/GTT	intron				7	7.11E-03	0.891	68	5.63E-01	0.071
VCR	21	30714976	-	*BACH1*	C/G	exon	A678G	Tolerated	Benign	10	3.71E-05	0.945	72	9.04E-01	0.015
	12	75816816	rs59277111	*GLIPR1L2*	-/CAA	exon	D239_K240insQ	NA	NA	10	6.11E-04	-0.888	76	8.66E-03	-0.299
	10	97397087	-	*ALDH18A1*	A/C	exon	V26G	Deleterious	Possibly damaging	6	7.47E-04	0.978	51	7.94E-01	-0.037
	19	56001803	rs5828624	*SSC5D*	-/CCAAGCAA	intron				9	9.35E-04	-0.900	72	6.99E-01	-0.046
	2	47277207	rs71416119	*TTC7A*	CA/AG	intron				10	1.44E-03	0.859	76	3.70E-01	0.104
	15	78581889	-	*WDR61*	-/T	intron				10	2.46E-03	0.838	76	1.19E-02	0.287
	3	44803116	rs3082548	*KIAA1143*	AAGACAG/-	5'UTR				10	3.69E-03	-0.820	76	2.53E-01	-0.133
	8	145692652	-	*KIFC2*	A/G	exon	S133G	Tolerated	Benign	7	4.86E-03	0.907	48	4.39E-01	0.114
	5	1246263	-	*SLC6A18*	-/GCCCCC	3'UTR				10	4.99E-03	0.805	76	4.36E-01	0.091
	12	29908581	rs3830194	*TMTC1*	-/TTGTT	intron				10	6.95E-03	-0.787	75	3.31E-01	-0.114
	7	100361392	rs3215395	*ZAN*	-/C	intron				10	7.45E-03	0.783	76	4.66E-01	0.085
	6	32713619	rs146449814	*HLA-DQA2*	C/A	exon	P128H	Deleterious	Probably damaging	9	8.51E-03	0.807	62	6.81E-01	0.053
	8	134292515	-	*NDRG1*	-/G	intron				10	8.87E-03	-0.772	76	8.60E-01	0.021
	1	203816588	-	*ZC3H11A*	T/A	exon	I440N	Tolerated	Benign	5	9.01E-03	0.962	50	2.41E-01	0.169
	9	138523408	rs34000956	*GLT6D1*	-/T	intron				10	9.25E-03	-0.770	76	6.23E-01	0.057
VLB	6	33037639	rs386699859	*HLA-DPA1*	GC/AT	exon	A42M	NA	NA	10	6.37E-04	0.886	76	1.96E-01	0.150
	6	32948287	-	*BRD2*	TT/GC	intron				10	6.49E-04	0.886	76	6.54E-01	0.052
	15	89864317	rs2307433	*POLG*	-/CTAC	intron				10	3.72E-03	-0.819	76	2.01E-01	0.148
	11	94322352	rs386756343	*PIWIL4*	AG/TA	exon	Q327L	NA	NA	10	4.26E-03	-0.813	76	4.89E-01	0.081
	20	62492851	-	*ABHD16B*	G/C	5'UTR				8	4.66E-03	0.873	65	8.77E-01	0.020
	12	16055927	rs71042275	*STRAP*	-/T	3'UTR				10	5.38E-03	-0.801	76	3.62E-01	0.106
	2	211421454	-	*CPS1*	-/CTT	exon	I5_K6insL	NA	NA	10	6.08E-03	0.794	79	7.54E-01	0.036
	1	9324725	-	*H6PD*	C/A	exon	P736T	Deleterious	Probably damaging	5	8.08E-03	0.964	45	7.12E-01	0.057
	21	47754410	rs57603484	*PCNT*	A/G	exon	S5G	Tolerated	Possibly damaging	9	9.17E-03	0.803	75	1.77E-01	0.158
	15	89864318	-	*POLG*	-/TACC	intron				10	9.98E-03	-0.765	76	4.25E-01	0.093

^a^ Based on GRCh37 genome assembly.

^b^ rsID from the NCBI database of genetic variation (dbSNP). "-", this variant is not identified in dbSNP.

^c^ Spearman correlation coefficient had been calculated to estimate positive (resistant) or negative (sensitive) correlation between the VAF and sensitivity to each anticancer drug.

5FU, 5‑fluorouracil; ACNU, nimustine; ADR, adriamycin; CPM, cyclophosphamide; DDP, cisplatin; MMC, mitomycin C; MTX, methotrexate; VCR, vincristine; VLB, vinblastine; ncRNA, noncoding RNA; del, deletion; ins, insertion; fs, frameshift.

**Table 12:** Variants associated with chemosensitivity to each drug (P < 0.01) in gastric cancer xenografts.

Drug	Chr	Position^a^	rsID^b^	Gene	Allele Ref./Variant	Location	Amino acid change	Prediction of functional effect		Gastric cancer			All xenografts		
								SIFT	PolyPhen2	N	*P* value	*r*_*s*_^c^	N	*P* value	*r*_*s*_^c^
5FU	8	19682402	-	*INTS10*	C/A	exon	Q309K	Deleterious	Possibly damaging	10	2.77E-03	-0.833	59	7.29E-02	0.235
	13	25466771	-	*CENPJ*	-/C	intron				12	4.73E-03	-0.753	76	6.63E-01	0.051
	6	153312232	rs149540839	*MTRF1L*	-/ATATG	intron				11	5.42E-03	-0.771	75	5.29E-01	-0.074
	17	21203998	rs62057674	*MAP2K3*	G/A	intron				12	5.80E-03	0.741	76	3.56E-01	-0.107
	2	113953976	rs1553408097	*PSD4*	CA/TG	intron				12	6.43E-03	-0.735	76	1.54E-01	-0.165
	6	33037639	rs386699859	*HLA-DPA1*	GC/AT	exon	A42M	NA	NA	12	9.22E-03	0.713	76	3.34E-02	0.244
ACNU	16	70287173	-	*AARS*	-/G	exon	Q907Pfs*23	NA	NA	12	2.32E-03	0.788	77	7.85E-01	-0.032
	19	17946871	rs397839895	*JAK3*	-/G	intron				12	4.51E-03	-0.755	77	2.98E-01	-0.120
	14	93399168	-	*CHGA*	-/A	exon	E271Gfs*19	NA	NA	12	5.36E-03	-0.746	77	4.75E-01	-0.083
	8	33451023	-	*DUSP26*	-/T	intron				12	6.54E-03	-0.734	77	4.03E-01	-0.097
	1	10384177	rs3831405	*KIF1B*	-/TTGAAA	intron				12	6.68E-03	-0.733	77	4.47E-01	-0.088
	7	21659555	rs57952953	*DNAH11*	-/TTAAT	intron				12	6.79E-03	-0.732	76	8.74E-01	-0.018
	4	48178004	rs11282767	*TEC*	-/AATCAGCC	intron				12	9.20E-03	0.713	77	4.10E-02	0.233
	12	11215037	-	*PRH1-PRR4*	-/A	ncRNA_intron				12	9.68E-03	0.710	77	5.13E-02	0.223
ADR	19	36290965	-	*PRODH2*	G/C	exon	P529R	Tolerated	Benign	10	4.21E-03	0.813	68	6.80E-01	0.051
	1	212615872	-	*NENF*	-/C	intron				12	4.62E-03	-0.754	77	9.81E-01	0.003
	10	23393222	-	*MSRB2*	-/G	intron				12	9.29E-03	-0.713	77	1.40E-01	-0.170
CPM	8	103664311	rs36083487	*KLF10*	-/A	intron				12	1.01E-03	0.823	79	7.18E-01	0.041
	2	232087473	-	*ARMC9*	-/G	exon	I180Dfs*8	NA	NA	12	1.48E-03	-0.808	79	3.73E-01	-0.102
	2	29287938	-	*C2orf71*	-/TGC	intron				12	8.74E-03	0.717	79	3.15E-01	0.114
	17	71433759	-	*SDK2*	-/G	intron				12	8.82E-03	0.716	79	5.20E-01	0.074
	10	70652195	-	*STOX1*	-/T	intron				12	9.68E-03	0.710	79	8.40E-01	-0.023
DDP	1	28203133	rs774954578	*THEMIS2*	C/T	exon	C43C			12	1.94E-03	-0.796	77	9.65E-01	-0.005
	9	136246047	-	*STKLD1*	T/C	intron				8	5.43E-03	0.866	68	6.02E-01	0.064
	17	10209869	-	*MYH13*	C/T	exon	E1791E			10	5.56E-03	-0.799	53	4.45E-01	-0.107
	16	113639	-	*RHBDF1*	G/A	exon	S136S			8	6.95E-03	-0.854	50	7.22E-01	-0.052
	9	18927887	rs199938722	*SAXO1*	T/C	3'UTR				12	6.99E-03	-0.730	77	3.46E-02	-0.241
	20	1896059	rs386811663	*SIRPA*	GT/AC	exon	V132T	NA	NA	12	7.15E-03	0.729	77	4.27E-01	0.092
	12	53509339	rs34924760	*SOAT2*	GC/TT	exon	A202A			12	7.46E-03	0.726	77	5.29E-01	0.073
	19	40886465	-	*HIPK4*	T/G	exon	Y478S	Deleterious	Probably damaging	11	7.73E-03	-0.751	74	2.60E-01	-0.133
	19	45649504	rs72019726	*PPP1R37*	-/GTAA	intron				11	7.86E-03	-0.750	75	7.09E-02	-0.210
	5	147695284	rs3217238	*LOC102546294*	-/TCA	ncRNA_intron				12	8.07E-03	-0.722	77	6.63E-03	-0.307
	11	6555318	-	*DNHD1*	G/A	exon	E971E			8	8.29E-03	-0.845	61	2.79E-01	0.141
	9	131185358	rs56988335	*MIR1268A*	-/TGTCCACTG	ncRNA_intron				12	8.45E-03	0.719	77	5.50E-01	0.069
	14	93399168	-	*CHGA*	-/A	exon	E271Gfs*19	NA	NA	12	8.67E-03	-0.717	77	1.19E-01	-0.179
	20	1895950	rs386811661	*SIRPA*	CCT/GTC	exon	D95_L96 delinsES	NA	NA	12	9.07E-03	0.714	77	3.30E-01	0.113
	8	33451023	-	*DUSP26*	-/T	intron				12	9.98E-03	-0.708	77	1.48E-01	-0.166
MMC	19	48282078	rs34940677	*SELENOW*	-/GCAGCGG	intron				10	3.63E-03	-0.821	75	2.33E-01	-0.139
	7	23293095	-	*GPNMB*	-/A	intron				12	4.73E-03	0.753	79	7.34E-01	-0.039
	15	52901283	rs386783993	*FAM214A*	TT/CC	exon	T617A	NA	NA	12	5.87E-03	0.741	79	7.36E-01	0.039
	1	158533221	-	*OR6P1*	-/G	exon	M59Hfs*32	NA	NA	12	6.62E-03	-0.734	79	3.40E-01	-0.109
	19	9000065	-	*MUC16*	-/T	intron				12	6.62E-03	0.734	79	6.42E-01	-0.053
	5	134210196	-	*TXNDC15*	G/T	exon	G27X	NA	NA	9	7.00E-03	-0.818	59	3.45E-01	-0.125
	10	135368490	rs3831169	*SYCE1*	-/GCTGAGACGG	intron				12	8.31E-03	-0.720	79	9.80E-01	0.003
	10	135368491	-	*SYCE1*	-/CTGAGACGGG	intron				12	8.31E-03	-0.720	79	9.44E-01	0.008
	8	11705381	rs145929462	*CTSB*	-/AGCCCCAGCT GGGCGAGGC	intron				12	8.82E-03	0.716	77	8.06E-01	0.028
	6	32362702	rs28362676	*BTNL2*	TG/CT	exon	P393Q	NA	NA	12	8.82E-03	-0.716	79	4.25E-01	-0.091
	15	101606889	rs386787404	*LRRK1*	GC/AA	exon	G1938E	NA	NA	12	9.59E-03	-0.711	79	7.75E-01	0.033
MTX	2	113953976	rs1553408097	*PSD4*	CA/TG	intron				11	1.39E-03	-0.835	68	9.03E-02	-0.207
	1	10384177	rs3831405	*KIF1B*	-/TTGAAA	intron				11	1.58E-03	-0.830	68	9.54E-01	0.007
	2	128878011	-	*UGGT1*	-/A	exon	V320Gfs*20	NA	NA	11	2.13E-03	0.817	68	1.86E-01	0.162
	15	101606889	rs386787404	*LRRK1*	GC/AA	exon	G1938E	NA	NA	11	2.38E-03	-0.812	68	3.77E-02	-0.253
	5	140203493	-	*PCDHA5*	G/A	exon	V711V			6	3.01E-03	-0.955	51	5.76E-01	0.080
	2	69597065	rs57860122	*GFPT1*	-/A	intron				11	3.12E-03	0.800	68	3.87E-01	0.107
	2	74642267	rs768089535	*C2orf81*	-/GCGGAGGGGCGG GTGGCGCCGCCC	exon	A251delins GAAPPAPPP	NA	NA	11	3.70E-03	-0.791	68	6.82E-01	-0.051
	16	81242149	rs796089514	*PKD1L2*	TTT/-	exon	N236del	NA	NA	11	4.96E-03	-0.776	68	4.89E-01	-0.085
VCR	17	21215682	rs73302038	*MAP2K3*	G/A	intron				12	8.32E-06	0.935	76	6.89E-01	0.047
	17	21215637	rs66486636	*MAP2K3*	G/A	intron				12	1.08E-05	0.931	76	3.72E-01	0.104
	17	21215700	rs73302043	*MAP2K3*	T/G	intron				12	1.73E-05	0.924	76	7.27E-01	0.041
	17	21215643	rs73302034	*MAP2K3*	A/G	intron				12	2.67E-05	0.917	76	4.27E-01	0.092
	21	47985555	rs74854320	*DIP2A*	-/G	intron				12	1.15E-03	-0.818	76	7.77E-01	-0.033
	6	79595168	rs66520304	*IRAK1BP1*	-/CTTAT	intron				10	1.43E-03	-0.859	71	5.47E-01	-0.073
	19	8808938	-	*ACTL9*	-/G	exon	G39Rfs*15	NA	NA	12	1.73E-03	-0.801	76	6.73E-01	-0.049
	7	100853907	-	*PLOD3*	-/C	exon	D470fs*0	NA	NA	12	4.76E-03	0.752	75	1.11E-01	0.186
	15	65931909	rs111310111	*SLC24A1*	-/CTGAGGC	intron				12	4.96E-03	0.750	76	5.75E-01	0.065
	5	180687440	rs3073543	*TRIM52*	TTC/-	exon	E130del	NA	NA	12	5.19E-03	-0.748	76	5.17E-01	-0.076
	3	49148887	-	*USP19*	-/C	intron				12	8.78E-03	-0.716	76	7.37E-01	-0.039
	8	142231944	rs386730897	*SLC45A4*	GC/AG	intron				12	9.39E-03	0.712	76	9.53E-01	-0.007
	1	203137787	-	*MYBPH*	-/CT	exon	X478delinsX	NA	NA	12	9.80E-03	-0.709	76	9.35E-01	-0.010
VLB	2	113953976	rs1553408097	*PSD4*	CA/TG	intron				12	2.29E-03	-0.789	79	4.25E-01	-0.091
	11	60617832	-	*CCDC86*	-/A	3'UTR				12	3.43E-03	-0.769	76	4.23E-02	-0.234
	19	45649504	rs72019726	*PPP1R37*	-/GTAA	intron				11	5.21E-03	-0.774	74	6.44E-01	-0.055
	13	98896915	-	*FARP1*	T/G	exon	S114R	Deleterious	Benign	7	7.37E-03	-0.889	53	5.46E-01	-0.085
	6	44122422	rs10537719	*TMEM63B*	CCT/-	intron				12	8.64E-03	0.717	76	8.18E-01	0.027
	1	10384177	rs3831405	*KIF1B*	-/TTGAAA	intron				12	9.55E-03	-0.711	76	9.29E-01	0.010

^a^ Based on GRCh37 genome assembly.

^b^ rsID from the NCBI database of genetic variation (dbSNP). "-", this variant is not identified in dbSNP.

^c^ Spearman correlation coefficient had been calculated to estimate positive (resistant) or negative (sensitive) correlation between the VAF and sensitivity to each anticancer drug.

5FU, 5‑fluorouracil; ACNU, nimustine; ADR, adriamycin; CPM, cyclophosphamide; DDP, cisplatin; MMC, mitomycin C; MTX, methotrexate; VCR, vincristine; VLB, vinblastine; ncRNA, noncoding RNA; del, deletion; ins, insertion; fs, frameshift.

## Discussion

Precision medicine demands the development of biomarkers to detect patient chemosensitivity to anti-cancer drugs. Here, we sought to identify clinically useful genetic markers for chemosensitivity to one or more of nine cytotoxic anticancer drugs by whole-exome sequencing for 79 xenografts. Although none of the genetic variants achieved a significance level after Bonferroni correction for multiple testing (*P* = 1.11 × 10^−6^), numerous variants showed possible associations with chemosensitivity to each of the nine tested drugs. Moreover, the subgroup analysis indicated chemosensitivity markers specific for breast and gastric cancers. We propose that our method could contribute to the development and optimization of personalized chemotherapy regimens among patients with cancer.

In the whole-exome sequencing analysis of 79 xenografts, we found that, the variant chr8:g.22960701insC, located in *TNFRSF10C* and *LOC254896*, had the most significant (i.e., lowest) *P* value for its associated chemosensitivity to ADR (*P* = 7.15 × 10^−5^, *r*_s_ = 0.437, Table 3, Fig 1). Although the function of *LOC254896* remains to be clarified, the down-regulated expression [32,33] and hypermethylation [34,35] of *TNFRSF10C* in colorectal, prostate, and breast cancers has been reported previously. Additionally, in vitro experiments have suggested that an upregulation in *TNFRSF10C* in response to ADR treatment may induce resistance to ADR [36]. TNFRSF10C is reported to protect cells from TRAIL-induced apoptosis [37], and thus may be associated with resistance to ADR through these pathways.

A variant (chr15:g.63673951 insG) located in the 5’UTR of carbonic anhydrase 12 (*CA12*) was also associated with resistance to ADR (Table 3). CA12 is a membrane carbonic anhydrase and plays important roles in several physiological functions, such as acid-base balance and calcification [38]. A recent in vitro study showed CA12 overexpression in chemoresistant colon cancer cells expressing the drug efflux transporter P-glycoprotein (Pgp). Moreover, ADR chemosensitivity in tumors overexpressing both CA12 and Pgp can be increased using CA12 inhibitors [39]. Therefore, the chr15:g.63673951 insG variant may increase resistance to ADR by altering CA12 expression; further functional analyses would be required to verify this hypothesis.

Moreover, a variant (chr2:g.189916175 T>G) associated with resistance to MTX was located in exon 42 of collagen type V alpha 2 chain (*COL5A2*) (Table 7). COL5A2 is upregulated in colorectal and breast cancers [40,41] and is associated with poor clinical outcome and poor survival rates in bladder cancer [42]. Studies have suggested that collagen expression increases tumor drug resistance by inhibiting drug penetration into the cancer tissue and increasing cellular resistance to apoptosis [43]. As shown in Table 10, we identified genetic variants that could be associated with multi-drug resistance or sensitivity. Some of these genes may be involved in the proliferation and invasion of tumor cells; for example, *NYAP2* is reported to activate PI3K, Akt and Rac1, and mediates remodeling of the actin cytoskeleton [44], whereas *ZNF277* regulates cell migration and invasion through phosphatase and tensin homolog (*PTEN*) [45].

In the subgroup analysis using breast and gastric cancer xenografts, we identified possible tissue-specific biomarkers in the response to anticancer drugs; however, most of these variants showed weak or no association in the whole-group analysis. These results suggest a degree of tissue specificity in sensitivity to cytotoxic anticancer drugs. rs386792906 (chr16:g.81253642 AG>TC), which showed the strongest association with MTX chemosensitivity in breast cancer xenografts, was located in intron 1 of *PKD1L2*. PKD1L2 is a member of the polycystin protein family, and may function as a component of cationic channel pores [46]. According to a previous study using The Cancer Genome Atlas (TCGA) dataset, overexpression of *PKD1L2* mRNA is associated with improved prognosis in patients with breast cancer [47]. Although the functional association between PKD1L2 and MTX is unknown, this variant may be a useful marker for predicting sensitivity to MTX, and may act as an indicator of prognosis for breast cancer in the clinical setting.

We investigated the functional consequences of the associations between the top variants and the response to chemotherapy by interrogating the expression quantitative trait loci (eQTL) information in the Genotype-Tissue Expression (GTEx) database [48]. rs3830265, which showed the strongest association with sensitivity to VCR, was associated with the expression of *PRKCD* in the skin (*P* = 7.3 × 10^−7^) and esophagus (*P* = 1.7 × 10^−5^). Moreover, rs3842515, which showed the strongest association with sensitivity to ACNU in breast cancer xenograft, displayed a cis-regulatory effect on *CCDC82* expression in several tissues, including esophagus, thyroid, skin, and nerve (*P*_*min*_ = 2.3 × 10^−13^). However, the functional associations between these genes (*PRKCD* and *CCDC82*) and sensitivities to the aforementioned drugs or mechanisms of drug metabolism remain unknown and require further investigation.

There were several strengths and limitations in our study. The main strength of our study is that we sought to identify tissue-agnostic predictive markers for chemosensitivity to nine cytotoxic anticancer drugs. As we have entered a new era of precision medicine, tissue-agnostic cancer therapy will continue to grow and expand treatment options for patients with cancer [49]. In addition to our tissue-agnostic approach, we also performed subgroup analyses of breast and gastric cancers as a deeper understanding of the genomic profiles of specific tumor types is also important. There were several limitations in our study. First, the total number of xenografts and the total number of each tumor type are small, and there were differences in the numbers of tumor types. Therefore, our study is likely to be underpowered to detect statistically significant variants or perform a subgroup analysis for all tumor types. Second, the results need to be confirmed using a larger number of samples, along with a functional analysis of the identified genes.

In conclusion, using whole-exome sequencing and a PDX model, we identified 162 genetic variants as possible susceptibility factors for sensitivity to one or more of the nine tested cytotoxic anticancer drugs. This method and the results presented herein may contribute to the development of personalized treatments for the prescription of optimal chemotherapy regimens. Although the underlying mechanisms should be further investigated using a larger number of clinical samples and molecular analysis, we propose that our findings may help to contribute to understanding the mechanisms of chemoresistance and chemosensitivity, and aid in the improved prognosis and quality of life for patients with cancer.
